# Cyclopædia of Drug Pathogenesy

**Published:** 1885-06

**Authors:** Ad. Lippe

**Affiliations:** Philadelphia


### CYCLOPÆDIA OF DRUG PATHOGENESY.

AD. LIPPE, M. D., PHILADELPHIA.

This *Cyclopædia* is issued under the auspices of the British Homœopathic Society and the American Institute. It is edited by Richard Hughes, M. D., and J. P. Dake, M. D., with the aid of the following consultative Committee: Great Britain—J. Drysdale, M. D., R. E. Dudgeon, M. D., A. C. Pope, M. D.; United States—Conrad Wesselhœft, M. D., E. A. Farrington, M. D., H. R. Arndt, M. D.

The first part of this *Cyclopædia of Drug Pathogenesy* is before us. A criticism as to its matter and manner is solicited. Protests against Rules 6, 7, and especially 9 have been freely entered, and have been published in the medical journals. The Chairman of the Bureau of Materia Medica of the American Institute has repeatedly, nay, unceasingly, spoken of the unreliability of the homœopathic materia medica; has occupied much time in urging the necessity of purging said materia medica from unreliable matter; and among such chaff were especially placed the symptoms arising from proving of drugs above the fourteenth potency.

In 1847 honest doubters of the accuracy and reliability of *Hahnemann's Materia Medica* resolved, in Vienna, to re-prove Natrum muriaticum. They did their work honestly, and the learned editors of the *Cyclopædia of Drug Pathogenesy* would gain an immense amount of knowledge if they would read the *Natr-mur.* papers by Dr. Watzke, published in the fourth volume of the *Homœopathic Journal*, published at Vienna in 1848. They first made provings with the crude salt up to the thirtieth potency, then the highest potency in use. They published the day-books first, followed this up with a systematized statement of the symptoms and the confirmation of these provings by the clinical experiment; they then gave the characteristic indications for its use, and wound up with a statement of the duration of its action, related remedies, and its antidotes. The honest Dr. Watzke tells his colleagues, on page 251 of said journal: "Concerning the posology of this remedy (Nat-mur.), I find myself unfortunately—I say 'unfortunately' (*leider*)—compelled to declare myself in favor of the higher potencies, although I should have preferred to represent the general, ordinary view of the use of larger doses. The physiological experiments made with the kitchen-salt, as well as the predominating large number of the clinical experiments, speak clearly and decidedly in their favor."

Dr. Watzke would have preferred it much, had the experiments made by him and others resulted differently. He was wedded to large doses, and, like the majority of homœopathic physicians then, as at present, he was unwilling to give credit to Hahnemann as *the* creator of a reliable materia medica, and also as the expounder of the mode of applying medicines for the cure of the sick under the unerring Law of the Similars. He deserves much credit for his unselfish honesty.

In 1885 it was reasonable to expect "progress," but we have been "disappointed." What are we to do with this new opus? Of what possible use can it be to the homœopathic physician? Is the student of materia medica expected to burn his books and take up this new and novel opus? Has not all the work he is expected to do—viz.: to systematize the provings into a useful materia medica—been done by others long ago? Was it not well done by that indefatigable Hahnemann? by Jahr, by Hering, by Bœnninghausen, by Allen, and a host of diligent men? What will it help the healer if he turns to page 126 of this monstrous disappointment, where he is informed that the *focus* of the action of *Aconitinum* is the medulla about the roots of the pneumogastric, hypoglossal, and spinal accessory nerves?

On page 127 we are told Aconitine is a narcotico-acrid poison, whose irritating properties manifest themselves especially in the mucous membranes! Of what possible use can such twaddle be to the healer?

Page 136, the provings of Dr. Warren, reported by Dr. Payne, are credited to Dr. Hale, while they were published in the *North American Journal*, August, 1860.

Æthusa is a complete failure. We miss the provings of Petroz (*Bulletin d. l. Soc. Med. Hom.*, 1847); also the proving in the *Journal d. l. Soc. Med. Hom.*, 1850.

Also Attomyer's paper in the *Neue Archiv*, 1844. What will become of the unfortunate children who violently vomit *curdled milk* if trusted to the tender care of the compilers of this *Cyclopœdia?*

What were the motives? Who are the leading men in this case? Here we find—No. 1, Dr. Richard Hughes; No. 2, Dr. J. P. Dake. Dr. Richard Hughes has just accomplished his last glorious act, after discovering that that noble journal, *The British Journal of Homœopathy*, had been sailing under false colors for over forty years; that curantur is, was, and will be "all wrong, and should read curentur." Richard Hughes,

M. D., published in Boston a book under the title, *The Knowledge of the Physician,* a course of lectures delivered at the Boston University School of Medicine, May, 1884, dedicated to the Dean of said School, I. T. Talbot, M. D. On page 78 we find the following: "Here is a patient in the agony of angina pectoris; his heart is, as it were, compressed, his breathing almost impossible; his face is deadly pale, his surface cold, his pulse small and contracted. Your old-school colleague steps forward and, taught by Dr. Lauder Brunton, applies to his nostrils a few drops of the nitrite of Amyl. In less than a minute his face begins to flush, he warms up, he breathes freely, and the intolerable breast-pang is gone. This is beautiful practice. But is it Homœopathy? Nay; for let a healthy man inspire the same substance, and the effect will be, not the pallor and coldness and constriction, but the flush—the dilation of the imprisoned arteries—which delivered the sufferer. In all common sense and justice, therefore, this action should be ascribed to the second of those three *modi operandi* of medicine described by Hahnemann; it is enantiopathic, affording all the speedy palliation characteristic of such remedies while open to all their disqualifications and reproaches. * * * * * * You will not hesitate, therefore, to give credit to antipathy when you meet it, nor will you ignore any other curative applications of drugs because they seem to lie outside the homœopathic method."

The learned Doctor impliedly asserts that nitrite of Amyl cures angina pectoris; its application is beautiful practice, and must not be ignored because it seems to lie outside of the homœopathic method. The premises are "a fatal error," and here again we offer documentary evidence.

We turn for this purpose to the *Cyclopœdia of the Practice of Medicine,* by Ziemssen, Vol. XIV. Under Angina Pectoris, on page 53, we are told of nitrite of Amyl: "Probably the favorable effect may be confined to cases possessing the character of vascular spasm. As it so easily causes syncope, it should, if possible, be used with even greater care than in cases of hemicrania." We are further told, "the general treatment of this disease is more of a puzzle than even the symptomatic; *the remedies are many, the cures few.*"

The learned lecturer again indulges in the fatal error that Homœopathy ever and under any circumstances professes to treat forms of diseases. *Never.* Impliedly the learned lecturer denies that angina pectoris is amenable to the homœopathic treatment under the strict application of the only law of cure, and that in that disease the antipathic palliative treatment by the indiscriminate application of nitrite of Amyl to the nostrils of the sufferer is beautiful practice! The premises are a fatal error, and the deductions drawn from erroneous premises must naturally be also fatal errors. Under what possible logic can there be various laws of cure—various modes of applying curative remedies? As homœopathists, we are compelled to adhere always and under all circumstances to the Law of the Similars; the results will always justify the strict adherence to the application of an immutable law of nature. However much a healer may be tempted to resort to possible palliatives, he will surely fail to *cure* the sick, while this aim can and will be certainly accomplished if he remain true to himself and to the system he professes to practice. We herein speak advisedly; and the angina pectoris is no exception to the applicability of the law. The most severe and apparently hopeless cases of this certainly alarming disease have been *cured*—we say *cured, fully cured*—by the painstaking mode of selecting for the sick (not the disease) the most similar remedy, and by applying it with proper care. There is *never* a necessity to set aside the Law of the Similars. It is here in evidence that the learned lecturer introduced his hearers into the more easy practice followed by the *eclectics*. Whatever curative applications lie outside of the homœopathic method, let them lie there. If they are curative in fact, and not illusively curative, as are *all* palliatives, then they must be homœopathic. Has not Hahnemann shown conclusively that all *cures* have always been made and can never be made by any other means save under the immutable Law of the Similars. And why, then, return to eclecticism?

Editor No. 2 is Dr. J. P. Dake. Why does he also turn his back on Homœopathy and on Hahnemann's methods? There was a time when a Homœopathic Life Insurance Company was started at Cleveland. The worthy President of said Company, ignorant alike of the laws governing the healing art, insurance companies, and logic, proclaimed that the success of said company would secure also the success of Homœopathy. The laws governing all such companies were ignored, and the Company went down.

It has long been the aim of the ex-President to annihilate Homœopathy. Various plans had so far failed; the American Institute was not yet ready to drop its honorable name; the Milwaukee test fizzled out; the bold stand taken by the anti-Hahnemannians, when they arbitrarily proclaimed that there were no curative powers beyond the fourteenth potency, met with merited derision.

Finally, it came to pass that the stronghold of the healing art was to be taken by surprise. The distinctive feature of Homœopathy as taught by Hahnemann was its materia medica. This now came to be apparent to the unsuccessful men who wanted to pervert Homœopathy into eclecticism. Destroy that stronghold, and the School will be hopelessly eclectic. That is the motive of the men now insulting the profession by hurling at them the *Cyclopœdia of Drug Pathogenesy*. What is this opus? It is the merest trash; it is useless as a drug pathogenesy. The question is now, how will the American Institute receive this "abortion"? How will the profession at large treat this bantling? And what will the Old Guard do who still live and who have spent a long life full of sacrifices and hard work in the establishment of our healing art, who faithfully followed the master, and who, by their successes, convinced a large number of the intelligent portion of our great country that Homœopathy is a science and an art not surpassed by any system of medicine? Will the veterans submit to this last insult? And what failures will follow the application of eclecticism as advocated under the pretense of its being Homœopathy by these learned editors if an epidemic like the cholera visits our country? They have blabbed about a *revised* materia medica and give us no materia medica at all!

